# Is this time really different? Flight-to-safety and the COVID-19 crisis

**DOI:** 10.1371/journal.pone.0251752

**Published:** 2021-05-26

**Authors:** Celina Löwen, Bilal Kchouri, Thorsten Lehnert

**Affiliations:** Department of Finance, University of Luxembourg, Luxembourg, Luxembourg; University of Almeria, SPAIN

## Abstract

During periods of market stress, risk-averse investors reallocate their investments from stocks to gold in a bid to hedge risks. Market participants interpret the induced gold price increase as an indication of safe-haven purchases and a signal of increased uncertainty in the general economic and financial conditions, thereby causing higher gold price volatility. The aim of this paper is to analyze whether this flight to safety effect can be observed during the COVID-19 crisis, which is considered to be a one-of-a-kind crisis and obviously of different origin compared to previous (financial) crises. By examining the interactions between the (option-implied) volatilities of the stock market (VIX) and of the gold (GVZ) and oil (OVX) markets, the main findings indicate that there is a granger causality in general between the equity market and the gold as well as the oil market. During the COVID-19 crisis, a stronger influence of the equity market on the oil market can be observed. Based on symmetric causality tests that are typically employed in the literature, this cannot be observed for the gold market. However, once we control for asymmetric causal interactions, we find that positive shocks in VIX cause positive shocks in GVZ. Hence, the typical flight to safety effect, similar to the one observed during other (financial) crises can also be identified for the COVID-19 crisis. The causality between the equity and oil market is triggered by political factors as well as the economic impact of the crisis which induces a sharp drop in demand for oil.

## Introduction and aim of the paper

Since the outbreak of COVID–19, global markets have been shaken due to the impact of the pandemic on the economy. Travel bans, curfews and lockdowns have dragged the global economy into recession. The majority of SME’s, small businesses and startups are facing an existential crisis through unprecedent insolvency and behavioral changes in demand, but the same also applies on mature businesses (i.e., airlines, hospitality, and leisure), which are at risk of bankruptcy. Governments and central banks all over the world have launched stimulus packages to curb the economic damage caused by the pandemic. Obviously, the current downturn does not fit into any prior models. The coronavirus pandemic and resulting recession have severely amplified trends that were already at work in the global economy, but how it evolves from here is a big unknown. In this paper, we aim to understand the effect of the pandemic on markets and the change in investors’ behavior as we focus on the implied volatilities of the oil, the gold and the equity option markets. Expected volatility is crucial for investors as it influences their decisions on portfolio optimization, risk management, and hedging approaches, as well as how derivatives are priced in the market. Investigating the interactions among the expected volatilities of e.g., stock and gold can provide new insights into the risk-diversification benefits of flight-to-safety strategies. Empirical findings suggests that options might be important in price discovery; for example, abnormal options trading volume and order imbalance can predict future stock returns. Recently, using new empirical measures of information leadership, Patel et al. [1] find that the role of options in price discovery is up to five times larger than previously thought. Approximately one-quarter of new information is reflected in options prices before being transmitted to stock prices, with options playing a more important role in price discovery around information events.

Gold is not only considered a monetary asset, but also acts as a source of wealth. It is often looked at as a safe investment in times of crisis since it is considered a less risky investment than other assets, e.g., equity or other commodities. Gold is also used as a hedge to balance out losses in the equity market. As a low-risk investment asset, the implied gold options volatility is important to understand investors’ behavior during normal times and during times of market distress. Furthermore, the crude oil price has a significant impact on the economy since many companies and sectors use oil either in production or distribution. Oil is considered to be one of the most important commodities in the world. Changes in the oil price (or in the price of other crude oil derivatives) are therefore very relevant for investors behavior, it can cause uncertainty in the markets and destabilize the economy (Gokmenoglua and Fazlollahia [2]). Consequently, it is important to consider the implied volatility of crude oil options for this paper. The CBOE has introduced a number of forward-looking implied volatility indices, the most popular being the VIX or fear index (Bastâ and Molnár [3]).

This paper considers the implied volatility of stock options (VIX), which serves as an indicator of the uncertainty in the equity market (e.g., fear index). Some of the market-wide risks reflected in VIX are also risks for the gold market. The shift of investments from the equity market to the gold market in times of increased equity market volatility is known as the flight to safety effect. But how would an increase in VIX affect the volatilities of gold prices? Gold volatilities may rise due to the safe-haven and inflation-hedge properties of gold. As risk-averse investors reallocate their investments from stocks to gold in a bid to hedge risks, the price of gold will rise. Investors interpret this price increase as an indication of safe-haven purchases and a signal of increased uncertainty in the general economic and financial conditions, thereby causing higher gold price volatility. Hence, researchers find (i) positive spillovers from the stock market volatilities to gold volatilities, (ii) a rise in both gold returns and volatility in times of higher stock market volatility (inverted asymmetric volatility), (iii) cross-market hedging, e.g. price shocks in one asset market that can generate price shocks in a related non-shocked asset market due to the wealth and asset substitution effects and (iv) rising volatilities, along with the possible negative correlations in contemporaneous stock-gold returns, that lowers the risk of stock-gold portfolios. The aim of this paper is to analyze the interdependencies between the implied volatility changes in the oil price, the gold price and the equity market, and to establish whether the flight to safety effect can also be observed during the COVID-19 Crisis, which is considered to be a one-of-a-kind crisis and obviously of different origin compared to previous (financial) crises.

The paper is structured as follows. Section 2 provides an overview of the implied volatility indices of options for oil, gold, and stocks. Section 3 gives an overview of existing literature, highlighting the flight to safety effect analysis in the literature. In section 4 the data used for the empirical analysis of this paper is described and summarized. Furthermore, this section gives information on the method to identify flight to safety days. The identified flight to safety days are used for the analysis in this paper. Section 5 deals with the research methodology that is used to perform the analysis, first to ensure the stationarity of the data and then to find the granger causality between the mentioned implied volatility indices. Additionally, section 5 defines the regressions for the statistical tests. The results are described and interpreted in section 6. Section 7 summarizes and concludes.

## Indices of implied volatility at CBOE

The Chicago Board Options Exchange (CBOE) publishes various indices of implied volatility for different types of options in order to provide a measure of the market’s expectation of future volatility. The indices are calculated based on the options prices and are quoted in percentage points. The options considered for the indices have different underlyings, so the indices can be clustered into different groups. There are volatility indices on stock indices (U.S. and non-U.S.), for example the CBOE NASDAQ volatility index or the CBOE Emerging Markets ETF volatility index. Another group of implied volatility index is on interest rates, for example the CBOE Interest Rate Swap volatility index. Furthermore, there are indices of implied volatility on commodity-related ETFs, for example the CBOE Gold ETF volatility index, which will be covered in the next section in more detail. Then, there are indices on currency-related futures and ETFs as for example the CBOE/CME FX Euro volatility index and indices on single stocks, such as the CBOE Equity VIX on Amazon or Apple. There is also a volatility index on the CBOE volatility index, which is covering options on the S&P 500 index. It is called the CBOE VIX of VIX index (CBOE Volatility Indexes [4]). This paper focuses on the gold, the oil and the equity market, therefore the relevant volatility indices for these markets are discussed below in greater detail.

### CBOE Gold ETF Volatility Index (GVZ)

Gold options are options that have gold futures or physical gold as their underlying. Buying a call option on gold gives the holder of the option the right to buy gold at a pre-defined price, whereas the put option gives the holder the right to sell gold at a pre-defined price, before expiration of the option. In the United States, gold options trade on the Chicago Mercantile Exchange (CME). Volatility indices calculated from real options prices can be found on the website of the CBOE (CBOE Gold Volatility Index [5]).

Relevant for the gold options market is the CBOE Gold ETF Volatility Index (GVZ). It “measures the market’s expectation of 30-day volatility of gold prices by applying the VIX methodology to options on SPDR Gold Shares” (http://www.cboe.com/products/vix-index-volatility/volatility-on-etfs/cboe-gold-etf-volatility-index-gvz). The SPDR gold shares exchange-traded fund (GLD) reflects the spot price of gold less fund expenses and can therefore be taken as a reference for gold price movements. It is a fund of the Standard & Poor’s Depositary Receipts (SPDR) family and is administered by State Street Global Advisors. As most other ETFs it has not much administrative fees, which makes it very attractive to investors. However, the ETF has become one of the largest actors on the gold market, therefore critics fear that it can influence the market by buying and selling shares (Groth [6]).

Options on SPDR Gold Shares are American style options, which means they can be exercised at any day during their validity. The underlying are 100 shares of the GLD ETF. The GLD has an undivided interest in the SPDR Gold trust, whose main investment is in gold bullions. The physical location of the gold is in the HSBC bank in London.

The GVZ has been calculated and published by the CBOE since 2008, the possibility to trade on this index came in April 2011 ((CBOE To Launch Trading on CBOE Gold ETF Volatility Index Options (GVZ) On April 12—Second New Tradable Product On Volatility Of Active ETF Options [7]). The basis for the calculation of the GVZ are real-time bid-ask quotes of GLD options, which are then processed according to the VIX calculation methodology.

### CBOE Crude Oil ETF Volatility Index (OVX)

Oil options are options that have future contracts on crude oil as their underlying. In the United States they are traded on the New York Mercantile Exchange (NYMEX). The holder of a crude oil call option has the right to obtain a long position in a crude oil future; the holder of a crude oil put option has the right to obtain a short position in a crude oil future, both at the pre-defined strike price and before the expiration date of the option.

In comparison to gold futures, that have only one underlying, crude oil futures can have many different types of crude oil as their underlying. There are 3 main benchmarks that most of the crude oil types can be assigned to. Firstly, there is Brent Crude, which refers to oil produced from various oil fields in the North Sea. It is mainly used to produce gasoline or diesel fuel, etc. Transportation of this oil type is easier than for others because it is transported through underwater pipes. Then, there is West Texas Intermediate (WTI), which summarizes crude oil types extracted from wells in the United States. Since it is difficult to transport, because pipelines need to go overland, it is quite expensive to ship it to other parts of the world. Hence, it is the primary benchmark for U.S. oil consumption and trade. It is used mostly for gasoline refining. The third benchmark refers to oil from the Middle East and is called “Dubai/Oman”. It has a slightly lower quality than the other 2 benchmarks presented previously (it has a higher sulfur content and is “heavier”). Since this is the only oil from the Middle East for investors to invest in, it still represents an important benchmark. The main demand for this oil comes from Asia (Understanding Crude Oil Benchmarks and Classifications [8]). There are more than 100 other and less traded benchmarks for crude oil, which will not be considered for the scope of this paper.

At NYMEX, options for light sweet crude oil, belonging to the category of WTI, and options for Brent Crude are traded. Since light sweet crude oil seems to be more relevant for the U.S. market, the empirical part of the thesis will deal with options on light sweet crude oil futures. The implied volatility for these crude oil options is tracked in the CBOE Crude Oil ETF Volatility Index (OVX), which “measures the market’s expectation of 30-day volatility of crude oil prices by applying the VIX methodology to United States Oil Fund, LP options” (http://www.cboe.com/products/vix-index-volatility/volatility-on-etfs/cboe-crude-oil-etf-volatility-index-ovx).

The underlying fund (United States Oil Fund) is an ETF that holds futures contracts on light sweet crude oil WTI traded at NYMEX, which is exactly the type of crude oil this thesis intends to focus on. The intention of the fund is to track oil prices as close as possible, using the near-term futures contracts of West Texas Intermediate as a benchmark. The fund’s performance reflects the daily spot prices of these futures less USO expenses (USCF [9]). The OVX has been published for the first time in July 2008 by the CBOE. Options on the USO have been traded since May 2007. Since they quickly became one of the most actively traded options contracts, the OECD decided to track the implied volatility of these option prices closer (CBOE Holdings [10]).

### CBOE Volatility Index (VIX)

As described earlier, equity options can be options on stocks or options on stock indices. The most relevant stock index for the U.S. market is the S&P 500, which comprises the shares of the 500 largest U.S. publicly traded companies. Options on the S&P 500 index are traded at the CBOE with the tracker symbol SPX. They are European style options, which means the trading account is settled in cash rather than ETF shares. SPX options are traded with a multiplier of 100, which means that the value of one contract is 100 times the price of one option. The advantage of index options is that the risk the investor faces is very limited, the amount is as high as the option premium. For an index call option, the profit is potentially unlimited, while for an index put option the profit is capped at the index level less the option premium paid (Turner [11]).

The expected volatility of S&P 500 index option prices is tracked in the CBOE Volatility Index (VIX), which “is recognized as the world’s premier gauge of U.S. equity market volatility” (http://www.cboe.com/products/vix-index-volatility/volatility-on-etfs/cboe-crude-oil-etf-volatility-index-ovx). It shows market’s 30-day expected volatility, which serves as a tool to measure market uncertainty. Options and futures are traded on the VIX since 2006 and 2004, respectively.

The VIX is designed to provide up-to-date information on how the S&P 500 index will fluctuate in the near future (30 days) according to market expectations. This is done by using the midpoint of the real-time bid-ask quotes on SPX options, which is the equivalent of an option’s market price. The bid-ask quotes are measured and updated every 15 seconds between 02:15 am and 08:15 am, as well as between 08:30 am and 03:15 pm every day (timings are given for the central time zone, which is the time zone for Canada, the United States and Central America) (Cboe Global Indices, LLC, 2019).

## Literature on (causal) interactions between the gold, oil and equity market

The co-movements of commodity and equity prices has received a lot of attention in the literature. Typically, during financial turmoil, one observes large swings not only in equity prices, but also in e.g. gold and oil prices, because commodities can be considered as a reliable measure of value, especially in times of crisis. Researchers identified extreme downside risk (EDR) as a good indicator of extreme market returns. High-EDR assets generally exhibit high idiosyncratic volatility, high value-at-risk, large negative co-skewness, and high bankruptcy risk (Huang et al. [12]). Fernàndez-Avilés et al. [13] analyze the co-movement of commodity markets under extreme market conditions. In particular, they provide extreme downside risk co-movement maps of these markets during recent distress periods. They propose an expected shortfall-multidimensional scaling approach and find no clear risk co-movement patterns, nor spillover effects. Fernàndez-Avilés and Montero [14] study the relationships between international stock markets during a number of extreme episodes. They construct financial maps by means of the multidimensional scaling technique and use spatio-temporal geostatistics to model the dependencies in stock market returns. Their results indicate that this combined methodology captures the propagation of returns in crashes better than in booms. Typically, most of the literature on price co-movements focuses on price levels, and much less attention is given to causal interactions in conditional volatility. Market interactions in terms of conditional volatility can provide better insights into the dynamic price relationships of markets (e.g. Gallagher and Twomey [15] and Gardebroek, et al. [16]. In general, it is known that in case of rising uncertainty in the stock, oil or gold market, stock prices fall. Uncertainty in the stock market is usually priced into the expected returns across the stock market (Bali and Zhou [17]). Empirical literature also suggests that when stock market uncertainty increases, as reflected in the market volatility index (VIX), bond returns rise while stock returns fall (Connolly et al. [18]), treasury and investment-grade bond yields fall (Jubinski and Lipton [19]), and one observes more flight-to-safety episodes (Baele, et al. [20]), which can be anticipated (Thomas [21]). On the other hand, oil market uncertainty seems to be sector-specific and only related to oil-relevant industries, which means it can be diversified away. Gold market uncertainty is only asset-specific, it relates only to investors that actually hold gold assets (Bams, Blanchard, Honarvar and Lehnert [22]). These statements imply that stock market uncertainty is most likely to influence other markets, whereas it seems less likely that the equity market is influenced by oil market uncertainty and gold market uncertainty. Gokmenoglu and Fazlollahi [2] analyze how equity prices are influenced by gold price changes, the oil price, as well as the implied volatility of oil (OVX) and the implied volatility of gold (GVZ). Using an autoregressive distributed lag model (ARDL), they investigate the long-term relationship of the variables. They find that an increase in the oil price has a significant influence on the stock market price, because many companies in the S&P 500 are somehow dependent on oil, for example for their production and for the distribution of their products. Investors see gold as a substitute investment for the stock market, a safer investment that can be used to hedge risks.

Sarwar [23] investigates the flight-to-safety effect using implied volatilities. This effect describes the behavior of investors in case of increased stock market volatility. They tend to invest in other markets like for example the gold market which is generally considered as a safe haven. The consequence is an increase of the gold price, acting as a potential signal for a general uncertainty in economic and financial conditions. This can lead to an increase in gold price volatility. The results suggest a positive effect of changes in VIX on changes of volatilities in the other markets. The flight-to-safety effects can be observed more intensely in times of crisis. The flight-to-safety effect is considered as an effect in cross-market hedging, as risk-averse investors re-balance their portfolios in times of crisis, focusing more on less risky assets like gold, silver and T-notes (Sarwar, 2016).

The traditional representative agent consumption-based asset pricing literature (e.g. Barsky [24] and Bekaert et al. [25]), defines flight-to-safety as the joint occurrence of higher economic uncertainty with lower equity prices and low real rates. In Vayanos [26], fear raises investors’ effective risk aversion, and the resulting flight-to-safety pushes up risk premiums and drives down the prices of risky assets. In Caballero and Krishnamurthy [27], flight-to-safety occurs in periods of low liquidity, when Knightian uncertainty leads agents to sell risky assets in favor of safer claims. In the theoretical model of Brunnermeier and Pedersen [28], flight-to-quality occurs, when speculators face margin requirements that increase in asset price volatility. Once they are hit by a negative shock, this can cause a liquidity spiral, with liquidity deteriorating across markets. Empirically, Baur and Lucey [29] find that gold is a hedge and a safe haven. They define a safe haven asset as an asset that “reduces losses in times of market stress or financial crisis by more than hedge or diversifier assets”. The results indicate that gold serves as a safe haven after severe negative stock market shocks, which means that stock market participants tend to invest their money in gold in times of stock market crisis. The study implies as well that investors sell their gold investments again after a decrease of volatility in the stock market. Bastâr and Molnár [3] investigate the correlation between the implied volatility of the stock market, measured by VIX, and the implied volatility of the oil market, measured by OVX by using the wavelet method. They find that VIX and OVX are strongly correlated, but not equally over different time periods. Furthermore, the results of their tests indicate that the VIX slightly leads OVX, which may be due to the fact that the underlying options used for VIX calculation are more liquid than the ones used for OVX calculation and the VIX therefore reacts faster to market factors. The authors imply that the diversification benefits of investors exposed to oil and stock market volatility are greater in the short run (a few days) than they are in the long run (a few years).

Wang and Chueh [30] find that crude oil prices and gold prices are connected through interest rates. Their results show that in the short run an increase in interest rates leads to falling gold prices, but rising crude oil prices, causing an increase in volatility in both markets. In the long term, interest rates lead gold prices, because they affect investors’ expectations of the US Dollar. If it decreases investors will move their capital to the gold market, which also represents a kind of flight to safety aspect and causes gold market volatility to rise. Compared to Wang and Chueh, our paper shows the direct interconnection between the implied equity market volatility, oil market volatility and gold market volatility.

## Data

The following section describes the data that is used for the empirical analysis of this paper. It includes time series of VIX, GVZ and OVX to represent the equity market, the gold market, and the oil market. This paper mainly focuses on market developments in the U.S., since the S&P 500, which is based on U.S. companies, is the basis for the VIX calculation and West Texas Intermediate crude oil is the basis for the calculation of OVX. Accordingly, the flight to safety periods that have been identified relate to episodes, where the U.S. market has been in distress.

### Implied volatility indices: VIX, OVX, GVZ

The data used for the analysis in sections 5 and 6 has been downloaded from finance.yahoo.com. We investigate the implied volatilities of the oil, the gold and the equity market, and, therefore, focus on historical data from OVX, GVZ and VIX. Since data for the gold volatility index is only available as of 11th of March 2010, the same starting point is used for the other two indices. Data has been obtained up to February 26^th^ 2021 on a daily basis, considering only business days when options were traded on these indices. For the analysis, closing prices are used for each index. Table 1 presents summary statistics.

**Table 1:** Summary statistics full sample.

**Index**	**Mean (%)**	**SD (%)**	**Min (%)**	**Max (%)**	**Skewness**	**Kurtosis**
OVX	36.16	19.03	14.50	325.15	5.42	47.53
Change OVX	0.004	4.91	-90.61	130.22	5.68	325.95
GVZ	17.36	5.13	8.88	48.98	1.13	2.44
Change GVZ	0.000	1.13	-9.50	13.16	1.09	15.53
VIX	18.02	7.48	9.14	82.69	2.59	11.39
Change VIX	0.004	1.92	-17.64	24.86	2.36	31.21

From the summary statistics, it can be observed that the standard deviation is greatest for the OVX, as well as for the first differences of OVX, which means that this index is subject to large fluctuations. The standard deviation is lowest for the GVZ. The distribution of the GVZ prices, as well as the first differences of GVZ are distributed more symmetrically than the others, which can be seen from their rather small skewness values. However, none of the distributions is fully symmetric, they are all positively skewed, which means they have longer and fatter tails on the right side of the distribution. The kurtosis is especially high for the first differences of the closing prices of each index. This indicates that the number of extreme values is significantly higher than for a normal distribution. We conduct normality tests on the variables, which suggest that normality can be rejected in all cases. In our empirical analysis, we control for that feature of the data by implementing a bootstrap simulation approach to generate critical values that are robust to non-normality.

### Flight to safety days

In addition to the implied volatility data, this study also considers flight-to-safety (FTS) days as defined in Baele et al. [20]. The authors identify a FTS day when a large, positive bond return coincides with a large, negative equity return, when there are negative high-frequency correlations between the stock returns and the bond returns and additionally when there is high volatility in the equity market, indicating increased market stress. If these three criteria met, they define this episode as FTS. To identify the FTS episodes, a regime-switching model with three regimes (equity, bond and FTS) is used, where each regime variable can take on a value of 0 or 1, 1 indicating an FTS day. The switches from low (high) volatility to high (low) volatility are captured in a non-FTS-related jump term, which switches to 1 in case of an unexpected volatility change in the equity or the bond regime and is 0 otherwise. Another FTS jump term should highlight the effect of particularly pronounced returns on the first day of an FTS episode, it equals 1 on the first day and 0 otherwise. Additionally, 2 other models have been used to verify the results. The study shows that equity volatility is especially high during the FTS periods (95% higher on average), but also an increase in bond volatility can be observed (23% higher on average). Another finding is that the correlation between stock and bond returns is dramatically lower (highly negative) in FTS periods and risk premiums are significantly higher, especially for equity. In general, FTS periods are short-lived, the observed maximum is 10 days, but they only rarely exceed 4 days.

In this paper, the goal is to investigate whether the observed interdependences between the three markets are different on the identified FTS days. Therefore, the data has been collected for the above-mentioned sample period relevant for this research. Hence, this allows for a test of the influence of VIX on GVZ and OVX only for FTS episodes.

## Research methodology

### Stationarity of the data

In order to ensure stationarity of the data, the first differences of the closing prices of the VIX, OVX and GVZ are considered for the further analysis. Given that we are focusing on a crisis period, potential structural breaks need to be considered when performing a unit root analysis. Kapetanios [31] provide tests for a unit root hypothesis against the occurrence of an unspecified number of breaks m. Results suggest that we can reject the null of a unit root at the 1% significance level for the three time series and all reasonable numbers of structural breaks m. We identify up to 3 break dates that are not related to the COVID-19 crisis period (25.11.2011, 24.08.2015 and 05.02.2018 for changes in VIX; 31.10.2011, 27.06.2013 and 10.02.2016 for changes in GVZ, and 25.11.2011, 12.02.2016; 23.11.2018 for changes in OVX)). This means that in all three cases the null hypothesis can be rejected, and the time series used for the further analysis is stationary.

### Granger causality—Wald test

The Granger causality test gives indication of links between random variables by using empirical data sets and finding correlation between these data sets. One condition of this concept is that the cause happens prior to the effect, which means that the time series for the cause variable need to be lagged. In order to perform a Granger causality test multiple time series of independent variables are needed, which are then tested for correlation (bottom-up approach). Using multiple time series (historical data sets) helps to understand the relationship between several components. A null hypothesis is established, which assumes that the development of the first variable does not cause a variation of the second variable. The goal of the Granger causality method is to provide a basis for a better forecast for the basis data, showing how other time series granger cause values in the time series of the basis data (Zaiontz [32]).

In our causality analysis, we also take into account other stylized facts of financial time series. We also investigate the potential asymmetric causal interaction between changes in the VIX and changes in the oil and gold implied volatilities. Given our focus on the flight-to-safety effects, we are particularly interested in the impact of positive changes in the VIX on changes in the oil and gold implied volatilities. Hatemi-J [33] and Hatemi-J and El-Khatib [34] suggest an approach that allows for asymmetry in the causality testing by using the cumulative sums of positive and negative shocks. However, asymmetric causality testing can also be implemented for stationary variables. In that case, positive or negative changes can be used instead of the cumulative sums. The authors show that the test can be implemented based on a Wald test statistic that is shown to follow a chi-square distribution asymptotically. However, we use a bootstrap simulation approach with leverage adjustment to generate critical values that are robust to non-normality (see Hatemi-J and El-Khati [34]), which is of particular concern given the data used in our analysis.

For the performance of the test, the correct number of lags relevant for the regression needs to be identified. The lag number indicates when the causality of the variables takes effect. The ideal number of lags can be identified using several information criteria. The most common ones are the Bayes Information Criterion (BIC), which is also called the Schwarz Information Criterion (SIC) and the Akaike Information Criterion (AIC). To find the optimal lag order, the value of the criterion should be minimized. For this time series, the SIC gives the most conclusive result, the optimal number of lags is found to be 4.

### Regressions

The granger causality test should provide insights of the predictive ability of the VIX on other non-equity implied volatilities, like GVZ and OVX, considering the lead-lag relationship between the first differences in VIX and the first differences of OVX and GVZ (Eq 2). Furthermore, it should also give results on whether non-equity implied volatilities have a predictive ability on the VIX, again considering the first differences of the indices (Eq 1). Finally, the granger causality test is used to define the additional lagged impact of the changes in VIX on the changes in GVZ and OVX during pre-defined flight to safety days (Eq 3). For these tests, the following predictive regressions can be estimated (Rapach et al. [35]):

$$\Delta_{VIX,t}=\alpha_{0}+\sum_{i=1}^{n}\alpha_{ns,i}\Delta V_{ns,t-i}+\sum_{i=1}^{n}\alpha_{VIX,i}\Delta_{VIX,t-i}+\varepsilon_{t}$$ (1)

$$\Delta V_{ns,t}=\beta_{0}+\sum_{i=1}^{n}\beta_{VIX,i}\Delta_{VIX,t-i}+\sum_{i=1}^{n}\beta_{ns,i}\Delta V_{ns,t-i}+e_{t}$$ (2)

$${\Delta V}_{ns,t}=\gamma_{0}+\sum_{i=1}^{n}\gamma_{VIX,i}\Delta_{VIX,t-i}+\sum_{i=1}^{n}\gamma_{ns,i}{\Delta V}_{ns,t-i}+\sum_{i=1}^{n}\gamma_{VIX\_FTS,i}\Delta_{VIX\_FTS,t-i}+u_{t}$$ (3)

$${\Delta V}_{ns,t}=\delta_{0}+\sum_{i=1}^{n}\delta_{VIX,i}\Delta_{VIX,t-i}+\sum_{i=1}^{n}\delta_{ns,i}{\Delta V}_{ns,t-i}+\sum_{i=1}^{n}\delta_{VIX\_Crisis,i}\Delta_{VIX\_Crisis,t-i}+\vartheta_{t}$$ (4)

Δ_*VIX*,*t*_ and Δ*V*_*ns*,*t*_ represent the changes (first differences) in VIX and the changes in implied OVX and GVZ (here grouped into the non-stock implied volatility). The changes of implied volatilities are measured at time *t*, *i* equals the number of lags used for this regression. As explained, the optimal number of lags has been determined as 4 as per the Schwarz Information Criterion.

For the Eq (1), $\sum_{i=1}^{n}\alpha_{ns,i}{\Delta V}_{ns,t-i}$ represents the lagged changes in OVX or ΔGVZ and $\sum_{i=1}^{n}\alpha_{VIX,i}\Delta_{VIX,t-i}$ represents the effect of the lagged changes of VIX on the changes of VIX at time *t*. This means that the VIX is not only tested for the influence of non-stock implied volatilities, but also for the influence of its own lagged values. *ε*_*t*_ is the error term in this regression. Eq (2) describes the influence of changes in VIX on non-stock implied volatilities OVX and GVZ using $\sum_{i=1}^{n}\beta_{VIX,i}\Delta_{VIX,t-i}$ as the lagged changes in VIX and $\sum_{i=1}^{n}\beta_{ns,i}{\Delta V}_{ns,t-i}$ as lagged changes of non-stock implied volatilities. *β*_0_ is the intercept and *e*_*t*_ is the error term in this regression. Eq (3) measures the additional influence of changes in VIX on changes in non-stock implied volatilities during FTS days, where $\sum_{i=1}^{n}\gamma_{VIX,i}\Delta_{VIX,t-i}$ is the effect of lagged VIX changes, $\sum_{i=1}^{n}\gamma_{ns,i}{\Delta V}_{ns,t-i}$ is the effect of lagged changes in non-stock volatilities and $\sum_{i=1}^{n}\gamma_{VIX\_FTS,i}\Delta_{VIX\_FTS,t-i}$ is the additional effect of lagged VIX changes on FTS day. In this regression *γ*_0_ represents the intercept and *u*_*t*_ the error term. Eq (4) measures the additional influence of changes in VIX on changes in non-stock implied volatilities during COVID-19 crisis days, where $\sum_{i=1}^{n}\delta_{VIX,i}\Delta_{VIX,t-i}$ is the effect of lagged VIX changes, $\sum_{i=1}^{n}\delta_{ns,i}{\Delta V}_{ns,t-i}$ is the effect of lagged changes in non-stock volatilities and $\sum_{i=1}^{n}\delta_{VIX\_Crisis,i}\Delta_{VIX\_Crisis,t-i}$ is the additional effect of lagged VIX changes on COVID-19 crisis days. In this regression *γ*_0_ represents the intercept and *δ*_*t*_ the error term.

## Results

In order to understand the data, it is helpful to look at the simple correlations of the variables in the regressions. The correlation matrix is presented in Table 2.

**Table 2:** Correlations between implied volatilities, full sample period.

Correlation	GVZ	OVX	VIX	dGVZ	dOVX	dVIX
GVZ	1.00					
OVX	0.44	1.00				
VIX	0.68	0.68	1.00			
dGVZ	0.11	0.00	0.08	1.00		
dOVX	0.03	0.13	0.08	0.16	1.00	
dVIX	0.02	-0.01	0.13	0.37	0.26	1.00

The correlations between GVZ and VIX and OVX and VIX are the highest, but there is also a significant correlation between GVZ and OVX. The correlations between the first differences are in general not as high as the correlations between the closing prices of the indices. All significant correlations are positive. The correlation matrix gives a good indication on the relationship between the indices. However, it does not provide any information on whether an implied volatility change is an effect of another implied volatility change. This will be further investigated in the next section.

### Causal interactions

In the following, we present the results from the symmetric and asymmetric Granger Causality tests between VIX, OVX and GVZ. The results obtained from the tests have been clustered into 3 sub-sections. First, the results of the full sample test are presented, then the findings from the full sample test with a focus on flight to safety days are discussed. Finally, the findings from the full sample test with a focus on the COVID-19 crisis period are presented.

### Full sample analysis

The first analysis to be done considers the whole test period from the 12th of March 2010 until February 26^th^ 2021. The results are summarized in Table 3.

**Table 3:** Causality tests.

Panel A: Symmetric Causal Interactions	
Test hypothesis	*Sum of coefficients*
dVIX does not Granger cause dOVX	0.63***
dOVX does not Granger cause dVIX	-0.02
dVIX does not Granger cause dGVZ	0.20***
dGVZ does not Granger cause dVIX	0.03
dGVZ does not Granger cause dOVX	0.89
dOVX does not Granger cause dGVZ	0.04
Panel B: Asymmetric Causal Interactions (Positive Shocks)	
Test hypothesis	*Sum of coefficients*
dVIX+ does not Granger cause dOVX+	0.37
dOVX+ does not Granger cause dVIX+	0.02
dVIX+ does not Granger cause dGVZ+	0.12***
dGVZ+ does not Granger cause dVIX+	0.18
dGVZ+ does not Granger cause dOVX+	0.41
dOVX+ does not Granger cause dGVZ+	0.02
Panel C: Asymmetric Causal Interactions (Negative Shocks)	
Test hypothesis	*Sum of coefficients*
dVIX- does not Granger cause dOVX-	0.43**
dOVX- does not Granger cause dVIX-	0.04
dVIX- does not Granger cause dGVZ-	0.18***
dGVZ- does not Granger cause dVIX-	0.08
dGVZ- does not Granger cause dOVX-	0.87**
dOVX- does not Granger cause dGVZ-	0.03***

Full sample 12.03.2010 to 26.02.2021. dVIX, dGVZ and dOVX represent the first differences calculated from the closing prices of the volatility indices. dVIX+, dGVZ+ and dOVX+ (dVIX-, dGVZ- and dOVX-) are the related positive (negative) shocks (see Hatemi-J (2012) and Hatemi-J and El-Khatib (2016)).

*** and ** indicate statistical significance at the 1% and 5% level.

In Panel A, we show the results from the tests assuming symmetric causal interactions, while in Panel B and C, we present our findings assuming asymmetric causal interactions, positive and negative shocks, respectively. From Panel A, it can be observed that the null hypothesis can be rejected in two out of six cases. The sum of coefficients is negative for the effect of the implied volatility of the oil market on the implied volatility of the equity market. Hence, if the implied volatility in the oil market goes up, it goes down in the equity market. However, the test is not significant since the p-value is well above 5%. Additionally, the granger causality is also not significant for the impact of the implied volatility of the gold market on the equity market and of the oil market on the equity market.

The first significant result shows that the implied volatility of the equity market granger causes the implied volatility of the oil market. This finding is consistent with the results presented by Bastar and Molnár [3]. They also find that there is a strong effect of the equity market volatility on the oil market volatility, especially in the long run and that VIX leads OVX. Furthermore, given the financialization of the oil market, the oil market is more and more exposed to a variety of financial instruments like options, futures, and exchange traded funds. The OVX is an index displaying the implied volatility calculated from options on oil futures. Oil futures and options are not only used for portfolio diversification, but also for speculation. This might explain the spill-over effect of equity market implied volatility to oil market implied volatility.

Another significant result with a p-value of below 1% suggest that the changes in the implied volatility of the equity market granger cause changes in the implied volatility of the gold market. This is in line with the papers of Gokmenoglu and Fazlollahi (2015) and Sarwar [23]. Gold is in general perceived to be a safe asset to invest in. During periods of market stress, risk-averse investors reallocate their investments from stocks to gold in a bid to hedge risks. Market participants interpret the induced gold price increase as an indication of safe-haven purchases and a signal of increased uncertainty in the general economic and financial conditions, thereby causing higher gold price volatility. This is in line with the flight to safety effect. As mentioned earlier, the changes in the implied volatility of the gold market do not granger cause the changes in implied volatility of the stock market, the sum of the coefficients is very low and the p-value well above the 5% significance level. This is also consistent with the findings of Sarwar [23] that there is no “flight from safety” effect.

In Panel B and C, we present the results of the asymmetric causality tests. By separating between positive and negative shocks, we are in a better position to test for potential flight to safety effects. For example, market stress can be considered to lead to a positive shock in the VIX, while a negative shock can be associated with a recovery of the equity market. One can derive two interesting observations from those results. Firstly, the granger causality between the equity market and the gold market is driven by both, positive and negative shocks in the VIX. A positive shock in the VIX leads to higher gold market volatility, while a negative shock in the VIX lowers gold market volatility. Secondly, the granger causality between the equity market and the oil market is mainly driven by negative shocks in the VIX. A negative shock in the VIX lowers oil market volatility, while there is no effect for positive shocks in the VIX. The other causal interactions between the gold and oil markets are inconclusive.

### Analysis using flight-to-safety days

In order to differentiate the effect during ‘normal times’ and particular episodes of market stress, we replicate the analysis and additionally control for the flight-to-safety (FTS) days that can be identified according to Baele, et al. [20]. We restrict our attention to the causal interactions between the equity market and the gold or oil market. Therefore, the additional influence of the VIX on these flight to safety days is tested on the OVX and the GVZ. Prior research suggests that the impact of the equity market on e.g. the gold market is particularly pronounced during periods of market stress (e.g. Sarwar [23]). The sample period includes all business days from the 12^th^ of March 2010 to February 26^th^ 2021. During this period 1,7% of days can be identified as FTS days. The results are summarized in Table 4.

**Table 4:** Causality tests, FTS days.

Symmetric and Asymmetric Causal Interactions		
Test hypothesis	*‘Normal Times’*	*FTS days*
dVIX does not Granger cause dOVX	0.69**	0.63
dVIX+ does not Granger cause dOVX+	0.26	0.27
dVIX- does not Granger cause dOVX-	0.43**	-2.77
dVIX does not Granger cause dGVZ	0.15***	0.16
dVIX+ does not Granger cause dGVZ+	0.04	0.20***
dVIX- does not Granger cause dGVZ-	0.18	-1.75

Full sample 12.03.2010 to 26.02.2021: dVIX, dGVZ and dOVX represent the first differences calculated from the closing prices of the indices. dVIX+, dGVZ+ and dOVX+ (dVIX-, dGVZ- and dOVX-) are the related positive (negative) shocks (see Hatemi-J (2012) and Hatemi-J and El-Khatib (2016)).

*** and ** indicate statistical significance at the 1% and 5% level.

The results suggest that changes in the VIX significantly cause changes in the implied volatilities of the gold and oil market. However, according to the symmetric causality tests, the impact is not particularly pronounced on FTS days. But once we control for the sign of the shock, we find that positive shocks in the VIX, market stress, cause positive shocks in e.g. gold implied volatilities, which is particularly pronounced (highly significant) on FTS days. Hence, this is in line with the safe haven hypothesis and cannot be observed for negative shocks in the VIX. In line with this interpretation, we also do not find FTS effects for oil.

### COVID-19 crisis analysis

In the following, the same test as above will be performed only considering the COVID-19 crisis period. In retrospect, the COVID-19 pandemic started when the first cases of the new coronavirus were identified in the city of Wuhan, China in December 2019 (World Health Organization [36]) and the coronavirus has spread across the world with an enormous speed. However, financial markets have only been affected at a later stage. For the US, we have seen one of the quickest falls into a bear market in history (greater than 30% decline), with the VIX starting to increase on February 14th 2020 and the S&P 500 plunging in March 2020. In prior crises like the Dot-Com Bubble and the Global Financial Crisis, the bear markets came about much more gradually, over the course of half to a full year. We have seen large swings in gold prices, which kept on increasing and reaching a new high on August 6^th^ 2020, while the VIX remained at a high level. Therefore, in our analysis, we define the crisis period from February 14^th^ 2020 until August 6^th^ 2020. Hence, in the following, we replicate the analysis and additionally control for the COVID-19 crisis days. Table 5 summarizes the results.

**Table 5:** Causality tests, COVID-19 crisis period.

Symmetric and Asymmetric Causal Interactions		
Test hypothesis	*‘Normal Times’*	*Crisis Period*
dVIX does not Granger cause dOVX	0.34	1.91***
dVIX+ does not Granger cause dOVX+	-0.06	1.15***
dVIX- does not Granger cause dOVX-	0.02	1.36**
dVIX does not Granger cause dGVZ	0.15***	0.20
dVIX+ does not Granger cause dGVZ+	0.04	0.22***
dVIX- does not Granger cause dGVZ-	0.12***	0.16

Full sample 12.03.2010 to 26.02.2021: dVIX, dGVZ and dOVX represent the first differences calculated from the closing prices of the indices. dVIX+, dGVZ+ and dOVX+ (dVIX-, dGVZ- and dOVX-) are the related positive (negative) shocks (see Hatemi-J (2012) and Hatemi-J and El-Khatib (2016)).

*** and ** indicate statistical significance at the 1% and 5% level.

One striking result of the symmetric tests is that the granger causality of the VIX on OVX is significantly stronger during the Covid-19 Crisis period, while the causal interactions of the VIX and the GVZ is significant during ‘normal times’, but not particularly pronounced during the crisis.Therefore, based on symmetric causality tests that are typically employed in the literature, we would reject flight-to-safety effects during the recent crisis. However, once we control for the sign of the IV shock, we find that positive shocks in the VIX, e.g. market stress, cause positive shocks in e.g. gold implied volatilities, which is particularly pronounced (highly significant) during the period when the COVID-19 crisis hit financial markets. Hence, during the crisis period, the increased volatility in the equity market was transmitted into the gold market. This is in line with the safe haven hypothesis and cannot be observed for negative shocks in the VIX. During periods of market stress, risk-averse investors reallocate their investments from stocks to gold in a bid to hedge risks. Market participants interpret the induced gold price increase as an indication of safe-haven purchases and a signal of increased uncertainty in the general economic and financial conditions, thereby causing higher gold price volatility. We also replicate the analysis assuming a smaller time window (the quick fall into a bear market from February 14^th^ 2020 until March23^rd^ 2020), but the results are robust. This is in line with Sarwar’s [23] findings that the flight to safety effect intensified during the global financial crisis period, and in line with our previous analysis, where we are looking at FTS episodes. Indeed, except for a sharp drop in March 2020, the price of the SPDR gold shares ETF has raised during the first phase of the crisis hitting the highest price in history during August 2020. However, from August 2020 onwards, we observe a decline in gold prices. The drop in the gold price originates in the selling of huge amounts of gold, because investors were in the need of cash due to the economic consequences of the coronavirus. Many companies needed to be saved from insolvency and owners had to pay short term liabilities with other assets, since the generated income has decreased significantly for many businesses.

Based on our asymmetric causality test, we find similar effects for oil. Since the beginning of January 2020, the prices of the United States Oil fund (on which the OVX is based), have been declining steeply. However, on 28^th^ of April a sudden significant increase could be observed, and the prices have been rising again since. The reason was a material cut in oil supply in the U.S. during the month of April and an agreement of OPEC+ countries for the biggest oil supply cut in history as of May 1^st^, 2020. This led to a spike in OVX during end of April (IEA [37]). On the other hand, the equity market has had the most severe turbulences in March 2020, due to the economic impact of the coronavirus in Europe and the U.S. This During the period when the COVID-19 crisis hit financial markets, the increased volatility in the equity market was transmitted into the oil market. Furthermore, this effect was supported by the big drop in oil demand that was a result of the economic impact of the crisis. Due to the almost complete stop of air traffic and a significant reduction in other transportation due to global travel restrictions and border closings, the crisis caused the oil demand to fall significantly. This was intensified by the oil price war between Russia and Saudi Arabia, resulting in oil price drops also in the U.S. and OPEC countries. Therefore, the changes in oil market volatility have been caused by the collapse of the real economy, which is reflected in the increased equity market volatility, as well as by other political factors.

## Summary and conclusion

This paper aimed to answer the question of interconnectedness of the gold, oil and equity market with a focus on the flight to safety effect during a period when the COVID-19 crisis hit financial markets. The paper first explained the implied volatility indices published by the CBOE. Then, different findings on the relationships of the gold, oil and equity market are discussed. The data used for the empirical analysis of this paper was collected for a period March 12^th^ 2010 until February 26^th^ 2021. Daily closing prices of the VIX, OVX and GVZ, as well as an index on flight to safety days in the U.S. serve as the basis for the analysis. In our causality analysis, we take into account particular stylized facts of these financial time series. Next to standard symmetric causality tests, we also investigate the potential asymmetric causal interaction between changes in the VIX and changes in the oil and gold implied volatilities. Given our focus on the flight-to-safety effects, we are particularly interested in the impact of positive changes in the VIX on changes in the oil and gold implied volatilities. We use a bootstrap simulation approach with leverage adjustment to generate critical values that are robust to non-normality, which is of particular concern given the data used in our analysis. Additional tests have been conducted to analyze whether there is additional influence of the VIX on the GVZ and OVX on flight to safety days and during the period when the COVID-19 crisis hit financial markets.

Our main findings indicate that there is a granger causality in general between the equity market and the gold as well as the oil market. During the COVID-19 crisis, a stronger influence of the equity market on the oil market can be observed. Based on symmetric causality tests that are typically employed in the literature, however, this cannot be observed for the gold market. However, once we control for asymmetric causal interactions, we find that positive shocks in VIX cause positive shocks in GVZ. Hence, the typical flight to safety effect, similar to the one observed during other (financial) crises can also be identified for the COVID-19 crisis. The causality between the equity and oil market is triggered by political factors as well as the economic impact of the crisis which induces a sharp drop in demand for oil.
